# Social support and depressive symptoms: exploring stigma and self-efficacy in a moderated mediation model

**DOI:** 10.1186/s12888-022-03740-6

**Published:** 2022-02-15

**Authors:** Dong-Fang Wang, Ya-Nan Zhou, Yue-Heng Liu, Yu-Zhu Hao, Jun-Hong Zhang, Tie-Qiao Liu, Yue-Jiao Ma

**Affiliations:** 1 Department of Psychiatry and Psychotherapy, Klinikum rechts der Isar, School of Medicine, Technical University of Munich, 81675 Munich, Germany; 2 Department of Psychiatry, The Second Xiangya Hospital, Central South University, The China National Clinical Research Center for Mental Health Disorders, Chinese National Technology Institute of Psychiatry, Key Laboratory of Psychiatry and Mental Health of Hunan Province, No. 139, Middle Renmin Road, Changsha, Hunan 410011 P.R. China; 3 Department of Addictive Behavior and Addiction Medicine, Central Institute of Mental Health, Medical Faculty Mannheim, Heidelberg University, 68159 Mannheim, Germany

**Keywords:** Substance use disorder, Stigma, Perceived social support, Self-efficacy, Depressive symptoms, Moderated mediation model

## Abstract

**Background:**

Although some psychological processes, such as stigma and self-efficacy, affect the complicated relationship between social support and depressive symptoms, few studies explored a similar psychological mechanism among individuals with substance use disorders (SUDs). Hence, this research investigates the mediating effects of stigma and the moderating effects of self-efficacy among the psychological mechanism that social support affects depressive symptoms.

**Methods:**

The study included 1040 Chinese participants with SUDs and completed a series of self-report questionnaires. R software was used to organize and clean up data sets and analyze mediation and moderation effects.

**Results:**

The result showed that stigma partially mediated depressive symptoms, while self-efficacy moderated this relationship. More specifically, less social support increased depression symptoms by bringing about higher stigma. Besides, subjects with higher self-efficacy are less susceptible to stigma and therefore have mild depressive symptoms. Furthermore, clinical and theoretical implications are discussed in our study.

**Conclusions:**

Chinese SUDs patients’ depressive symptoms were indirectly affected by perceived social support via stigma and less affected by stigma with improved self-efficacy. The theoretical and practical implications of these results are discussed.

## Introduction

SUDs are a severe worldwide health problem, which places a major socioeconomic and public health burden on modern societies. According to the world drug report, nearly 271 million people have experienced drug use in 2018, and 35 million individuals suffered from SUDs [1]. Similarly, China has a long history of illicit drug use, with 2.14 million individuals suffering from SUDs, according to the latest survey in china [2].

People with SUDs are more likely to have a depression disorder, with some previous surveys have consistently reported high rates of comorbid SUDs and depressive symptoms [3, 4]. Individuals with alcohol or drug dependence were four and nine times more likely to suffer from major depression, respectively, than individuals with no substance dependence [5]. Such comorbid disorders cause serious clinical issues, as they have been linked to greater social and vocational impairment, relapse, poor treatment outcomes, higher morbidity, mortality, and more treatment costs [3, 4, 6–9].

Social support, one of the essential factors that can affect depressive symptoms of people with SUDs, is a concept that one feels cared for by others and has a reliable social network, such as family members, friends, and significant others [10]. Some previous studies demonstrate that perceived social support positively relates to psychological well-being [11–13], and protects against depressive symptoms and psychological distress [14–16]. For example, family support, such as administering medication, cooking meals, and emotional support, could help patients recover [17]. Conversely, lacking social support or suffering from social isolation will adversely affect mental health [18].

**Fig. 1:**
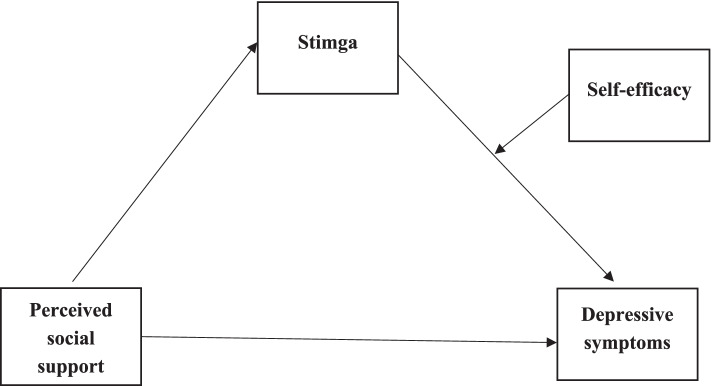
Conceptual framework of the current study

In summary, good social support can help reduce depressive symptoms among patients with SUDs. However, the study on how and when social support affects depressive symptoms in patients with SUDs remains unclear. Therefore, it is necessary to explore the relationship between perceived social support and depressive symptoms. With this contention in mind, we review the existing literature and propose a model depicted in Fig. 1.

### Hypotheses development

#### Perceived social support and stigma

At a personal level, stigma is a multifaceted construct and can be considered three separate but correlated constructs: Enacted, Anticipated, and Internalized stigma. Enacted stigma reflects past experiences of discrimination from others [19]. Anticipated stigma reflects individuals’ expectations about future discrimination. Internalized stigma is seen in endorsing and applying negative feelings and beliefs about people with SUDs to themselves.

Stigma is a mark of shame and disapproval resulting in an individual being rejected, discriminated against, and excluded from society [20]. People who have a characteristic that others discriminated (e.g., SUDs) will recognize themselves as inferior to others (of low social ranking) according to social rank theory [21]. Comparing to other mental illnesses, the general public holds a more serious stigma against people with SUDs [22], as they regard people with SUDs as violent and dangerous [23]. Nearly 58–78% UK population think that individuals with SUDs are dangerous, unpredictable, hard to talk to, and have themselves to blame [24]. The USA has reported a greater willingness to discriminate against SUDs individuals in terms of employment, housing, and governmental policy [22]. A systematic review including 28 studies showed that individuals with SUDs would experience discrimination not only from the public but from health professionals [25]. As a consequence, it would negatively affect treatment efficacy [25]. Therefore, they are more likely to experience severe social isolation in personal life or workplace and be treated differently in national policies [22, 26].

Significant others can work as discriminators or a source of social support [27]. Close others who find out about someone’s substance abuse treatment sometimes do not yet exert strong support support [28]. People with SUDs usually experience a serious stigma by significant-close others (e.g., family, partner, friends [29]). People with SUDs are highly susceptible to be shunned, insulted, marginalized, rejected, with stigma enhancing social exclusion in people who need the most social support [30].

Previous studies have indicated that social support was inversely associated with internalized stigma [31, 32]. Stigma has a negative impact on social support, but social support can positively affect stigma. For example, a poor social network has been shown to increase internalized stigma in schizophrenia [33]. Conversely, Poor interpersonal relationships may increase stigma among patients with HIV/AIDS [33, 34]. If people with SUDs feel supported by close others (e.g., relatives or friends), they could diminish internalized public views, thus lowed internalized stigma. Therefore, we hypothesize that perceived social support is negatively related to stigma. We hypothesize that perceived social support will be inversely associated with stigma (Hypothesis 1).

#### Stigma and depressive symptoms

We expect that stigma will positively associate with depressive symptoms. The stigmatization of individuals with SUDs may cause emotional, physical, cognitive stress responses [35], even mental disorders - depression and anxiety [36–41]. For example, a study about alcohol addiction showed that the feeling of hurt resulted from stigma would easily convert into depressive symptoms, such as unworthiness or embarrassment [42]. A similar conclusion has also been proved in patients with opioid use disorder [43]. Therefore, we hypothesize that stigma will be positively associated with depressive symptoms (Hypothesis 2).

#### The mediating role of stigma

Hypothesis 1 predicts a negative relationship between perceived social support and stigma, and Hypothesis 2 predicts a positive relationship between stigma and depressive symptoms. Together, these hypotheses specify a model in which perceived social support indirectly diminishes depressive symptoms by contributing to stigma. This notion is in line with Birtel et al. [44]; The mediation effect of internalized stigma between the perceived social support and depressive symptoms with a small size of 64 SUDs individuals, which indicated that if one with SUDs can feel more supported by others, such as family remembers or friends, they may lower be internalizing the negative public views to them and then reduce internalized stigma to reduce depressive symptoms. Therefore, we hypothesize that stigma will mediate the relationship between perceived social support and depressive symptoms (Hypothesis 3).

#### The moderating role of self-efficacy

Self-efficacy is a faith that an individual can successfully execute behaviors to achieve desired aims [45]. Self-efficacy could improve the ability to change substance use behaviors, such as drinking behavior [46, 47]. In addition, self-efficacy might contribute to cognitive and behavioral changes [48]. Although some studies have shown a correlation between self-efficacy and stigma in individuals with mental illness [49] and alcohol addiction [41], few studies have explored the relationship between stigma and self-efficacy in SUDs patients.

High self-efficacy is associated with positive outcomes, such as a low depressive symptom and increased well-being [50–52]. In addition, people with solid self-efficacy have good emotional regulation ability. Hence, they are more likely to perceive satisfaction and experience more positive emotions [53, 54]. In contrast, low self-efficacy is more likely to produce negative emotions, like depression, anxiety [55, 56]. Additionally, many studies have demonstrated that patients with depressive symptoms reported low self-efficacy [57–59]. Therefore, we speculate that self-efficacy may have a moderating effect on stigma and depression symptoms. Thus, we hypothesize that the positive relationship between stigma and depressive symptoms will be weaker for team high on self-efficacy than for team low on self-efficacy. Moreover, self-efficacy will moderate perceived social support’s positive and indirect effect on depressive symptoms (Hypothesis 4).

## Method

### Participants and procedure

It is a descriptive case-control study conducted at two Compulsory Drug Rehabilitation Centers in Hunan province, China. According to the Diagnostic and Statistical Manual of Mental Disorders, patients had to meet the diagnosis of substance use disorders (SUDs) according to the Diagnostic and Statistical Manual of Mental Disorders (DSM-5). The study was conducted from February 2020 until the end of December 2020. After signing the informed consent, subjects finished the relevant questionnaires. The investigation gained ethical approval from the second Xiang-Ya Hospital of Central South University (Application Number LYF2020109).

### Measures

#### Social-demographics questionnaire

The research team developed the Social-Demographics Questionnaire in light of the literature. It consists of eight questions to collect information about the participants’ socio-demographic characteristics, including age, job, gender, salary, marital status, education level, smoking and drinking, and substance use-related characteristics.

#### The Substance Use Stigma Mechanisms Scale (SU-SMS)

The Substance Use Stigma Mechanisms Scale measured stigma mechanisms among patients with SUDs [19]. This study used the Chinese version of SU-SMS (SUSMS-C) [60], containing five factors and 18 items on a five-point Likert scale (The total SUSMS-C score ranged from 18 to 90 points.). The higher the score on the scale, the more severe the stigma suffered by the individual. The SUSMS-C has good reliability, and validity in Chinese patients with substance use disorder, showing the internal consistency reliability is between 0.724–0.909, the test-retest reliability is 0.702 [60]. In the present research, Cronbach’s alpha was 0.88.

#### The multidimensional scale of perceived social support (MSPSS)

In this study, we used the Chinese version of the Perceived Social Support Scale (MSPSS) to assess the level of social support [61], which contains a total of 12 items on a seven-point Likert scale (The total MSPSS score ranged from 12 to 84 points.). Cronbach’s alpha of MSPSS was 0.89 in Chinese adolescents [61]. In the present research, Cronbach’s alpha was 0.92.

#### The Centre for Epidemiologic Studies Depression Scale (CES-D)

We assessed depressive symptoms with CES-D [62]. CES-D contains 20 items on a four-point Likert scale (The total CES-D score ranged from 0 to 60 points.). The higher the total score, the more severe the depression. This Chinese version of the CES-D has satisfactory reliability and internal validity and has been widely used in the Chinese population [63]. In the present research, Cronbach’s alpha was 0.87.

#### The General Self Efficacy Scale (GSES)

We used the General Self Efficacy Scale (GSES) [43], which contains ten items based on four responses, to assess self-efficacy (The total GSES score ranged from 0 to 40 points.). A lower score indicates a lower level of general self-efficacy. This scale has good reliability and validity in the Chinese population [64]. In the present research, Cronbach’s alpha was 0.71.

### Statistical analyses

We used R software (version 3.6.3) to organize and clean the dataset and generate the correlations. We used ‘***process*** [65] and the ‘***lavvan’*** packages [66], which allows us to analyze mediation and moderation effects at the same time [67]. We tested a moderated mediation model, in which social support served as the independent variable (X), negative affect stigma served as the mediating variable (W), depression served as the dependent variable (outcome, Y). Self-efficacy served as the moderator variable (V). Additionally, we also performed a simple slope computation of the moderation model to test the significance of the moderation slopes. To make the results more robust, we conduct the bootstrapping procedure [68, 69]. In this research, we adopt 1000 Bootstrap samples.

## Result

### Demography

One thousand and forty SUDs (204 females, 836 males) aged 16 and 65 years (Mean = 35.38, SD = 8.49) took part in this research. In this study, 785 participants (75.48%) mainly used methamphetamine, following heroin users were 171 (16.44%), and ketamine users were 62 patients (5.96%) (Table 1).

**Table 1:** Descriptive statistics of socio-demographic. (*N* = 1040)

	Total N (%)
**Gender**	
Male	836 (80.4%)
Female	204 (19.6%)
**Occupation**	
Unemployment	143 (13.75%)
Employment	897 (86.25%)
**Marry**	
Unmarried	630 (60.58%)
Married	410 (39.42%)
**Income (CNY/Month)**	
< 2000	153 (14.7%)
2000 ~ 5000	365 (35.1%)
5000 ~ 10,000	336 (32.3%)
> 10,000	186 (17.9%)
**Smoking**	
Y	987 (94.9%)
N	53 (5.1%)
**Drinking**	
Y	536 (51.5%)
N	504 (48.5%)
**Drug kind**	
Methamphetamine	785 (75.48%)
Heroin	171 (16.44%)
Ketamine	62 (5.96%)
Others	22 (2.12%)

### Correlations and regressions

Table 2 presents means, standard deviations, and intercorrelations for all variables. An inspection of the correlations reveals that the score of CES-D positively linked with that of SU-SMS (*r* = 0.493, *P* < 0.001), while negatively correlated with that of GSES (*r* = − 0.327, *p* < 0.001), and MSPSS (*r* = − 0.327, *P* < 0.001). Further analysis of the data revealed that there was a significant negative correlation between SU-SMS and GSES (*r* = − 0.155, *p* < 0.001), as well as MSPSS (*r* = − 0.273, *P* < 0.001). Additionally, MSPSS positively correlated with GSES (*r* = 0.293, *P* < 0.01).

**Table 2:** Means, standard deviations and correlations for the variables

	Means (SD)	GSES	CES-D	SU-SMS	MSPSS
**GSES**	24.020 (6.732)	1			
**CES-D**	20.920 (9.218)	−0.327**	1		
**SU-SMS**	45.180 (11.377)	−0.155**	0.493**	1	
**MSPSS**	58.750 (12.723)	.0293**	−0.333**	− 0.273**	1

*SD* Standard deviation, *SU-SMS* Substance Use Stigma Mechanisms Scale, *MSPSS* The multidimensional scale of perceived social support, *CES-D* Centre for Epidemiologic Studies Depression Scale, *GSES* General Self Efficacy Scale

* *p* < 0.01, ** *p* < 0.001

The result of linear regression analysis with depression as the dependent variable and clinical data (MSPSS, SU-SMS, GSES, GSES: MSPSS) as independent variables showed that MSPSS-C (β = − 0.113, *p* < 0.001), SU-SMS (β = 0.315, *p* < 0.001), GSES (β = − 0.327, *p* < 0.01), the interaction of GSES and MSPSS-C (β = − 0.015, *p* = 0.001) were independent variables to predict CES-D, and the total explanatory quantity of the three variables was 35% (Table 3).

**Table 3:** Regression results for simple mediation

Outcome	Predictors	Path	β	SE	t-value	P(>|z|)	LLCI	ULCI
**SU-SMS**	**MSPSS**	(a)	−0.244	0.027	−9.141	< 0.001	−0.296	−0.192
**CES-D**	**MSPSS**	(c)	−0.113	0.020	− 5.740	< 0.001	−0.151	− 0.074
	**SU-SMS**	(b1)	0.681	0.067	10.169	< 0.001	0.550	0.812
	**GSES**	(b2)	−0.361	0.127	2.842	< 0.001	0.112	0.610
	**SU-SMS: GSES (centralization)**	(b3)	−0.015	0.003	−5.400	< 0.001	−0.021	− 0.010

*SU-SMS* Substance Use Stigma Mechanisms Scale, *MSPSS* The multidimensional scale of perceived social support, *CES-D* Centre for Epidemiologic Studies Depression Scale, *GSES* General Self Efficacy Scale

### Tests of mediation

Table 3 presents the results for Hypotheses 1–3. Supporting Hypothesis 1, perceived social support was positively associated with stigma, as indicated by a significant unstandardized regression coefficient (β = − 0.244, t = − 9.141, *p* < 0.001, 95% CI [− 0.296, − 0.192]). Also, in support of Hypothesis 2, the positive relationship between stigma and depressive symptoms, controlling for perceived social support, was supported (β = 0.681, t = 10.169, *p* < 0.001, 95% CI [0.550, 0.812]). And finally, perceived social support has an indirect effect on depressive symptoms; this indirect effect was negative (− 0.077, 95% CI [− 0.102, − 0.055], when self-efficacy is normal), as hypothesis 3.

### Tests of moderated mediation

Table 4 presents the results for Hypotheses 4. We predicted that the inverse relationship between stigma and depressive symptoms would be weaker for teams high on self-efficacy than for teams low on self-efficacy. Results indicated that the cross-product term between stigma and self-efficacy on depressive symptoms was significant (β = − 0.015, Z = − 5.400, *p* < 0.001, 95% CI [− 0.021, − 0.010]).

**Table 4:** Conditional indirect effect of perceived social support on depressive symptoms through stigma by self-efficacy

Self-efficacy	Boot indirect effect	Boot SE	Boot LLCI	Boot ULCI
Low self-efficacy (the mean – 1 SD = 17.289)	− 0.102	0.026	−0.134	− 0.072
Moderate self-efficacy (the mean = 24.022)	− 0.077	0.022	− 0.102	−0.055
High self-efficacy (the mean + 1 SD = 30.753)	−0.052	0.0314	−0.075	− 0.033

**Fig. 2:**
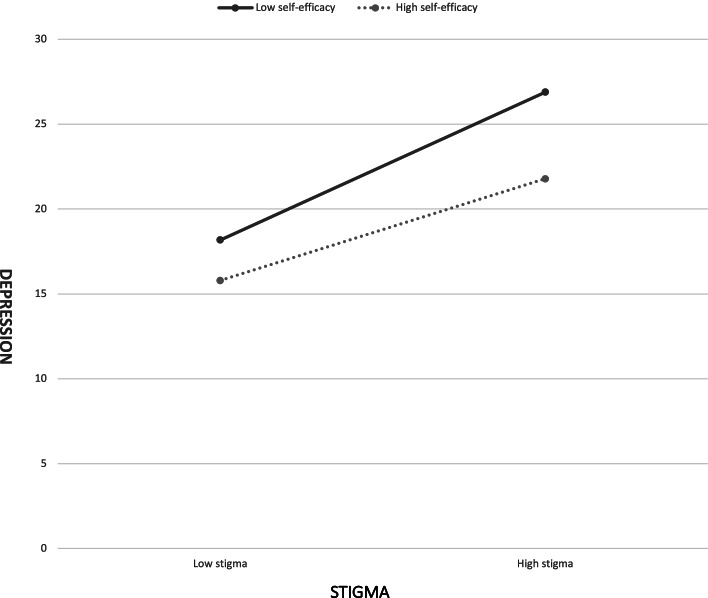
Moderation of the effect of Stigma on depressive symptoms by self-efficacy

We examined the conditional indirect effect of perceived social support on depressive symptoms (through stigma) at three values of self-efficacy (see Table 4): the mean (− 24.021), one standard deviation above the mean (6.732), and one standard deviation below the mean (− 6.732). Normal-theory tests indicated the three conditional indirect effects (based on moderator values at the mean and at 1 SD) were negative and significantly different from zero. Bootstrap CIs corroborated these results. Thus, Hypothesis 4 was supported. The indirect and negative effect of perceived social support on depressive symptoms through stigma was observed when levels of self-efficacy were low to high. Figure 2 shows the moderation effect of self-efficacy on the relationship between stigma and depressive symptoms.

## Discussion

The present study explored the mediator role of stigma between social support and depressive symptoms among SUDs patients by a moderated mediation model. We developed an integrated conceptual scheme that proposed that the relationship between perceived social support and depressive symptoms is more complex than previous research has indicated (e.g., Birtel et al. [44]). Initially, we predicted stigma to operate as a mediating mechanism between perceived social support and depressive symptoms. We then determined if self-efficacy could reduce the indirect relationship between stigma and depressive symptoms. Study results supported the hypothesized moderated mediation model, demonstrating that the magnitude of the indirect effect was contingent upon self-efficacy. This finding demonstrates the presence of a heretofore unidentified boundary condition influencing the impact of perceived social support on depressive symptoms.

We believe our results contribute to the literature by corroborating and extending prior findings in several ways. Previous studies devoted little attention to the relationship between perceived social support and depressive symptoms. To the best of our knowledge, no previous study has investigated the mechanisms connecting these constructs. The present study is the first to widen the focus of dysfunctional behavior research and present a more complex scenario of how perceived social support influences depressive symptoms. Based on the current results, SUDs individuals receiving less social support increased depressive symptoms by bringing about higher stigma. At the same time, SUDs individuals with a trait of high self-efficacy would be able to relieve depressive symptoms implications of stigma. This finding is important because it suggests that despite a strong relationship between perceived social support and stigma, the all-important second linkage between stigma and depressive symptoms diminishes when SUDs people’s self-efficacy is high.

An exciting finding indicated that the SUDs-related stigma mediates the relationship between perceived social support and depressive symptoms—in line with previous studies, suggesting that a poor social network could induce severe internalized stigma in schizophrenics [33, 34]. It has been widely reported that social support can be used as a predictor of depressive symptoms and stigma [14, 15, 31, 70, 71]. A study in a population of women infected with Acquired immunodeficiency syndrome (AIDS) found that stigma could mediate the relationship between social support and depressive symptoms [72]. Similar results were also found in substance abuse patients [44]. Therefore, the above results indicate that a supportive environment, including equal acceptance of SUDs patients and providing necessary help and care, can help patients build self-esteem and alleviate depression.

On the contrary, a hostile environment can cause SUDs patients to believe that they are primarily responsible for the disease, exacerbating the stigma. This finding highlights the importance of social support for SUDs patients. SUDs patients with reliable social support are accompanied by lower stigma, which can help reduce the mental stress associated with SUDs, such as depression symptoms. Our finding emphasized the necessity to provide more social support for SUDs patients and reduce their stigma. We also found that self-efficacy moderated the relationship between stigma and depressive symptoms. Although there have not yet been any studies investigating self-efficacy as a moderate factor among the patients with SUDs, a former study showed that stigma was significantly related to reduced drinking-refusal self-efficacy among individuals with alcohol addiction [41]. However, another study did not prove that self-efficacy could influence the stigma of internet addiction [73], which is inconsistent with our results. The discrepancy could come down to the different samples, as internet addiction is not regarded as a mental illness. We speculate that people with internet addiction would perceive less stigma from their family members and peers than patients with SUDs.

People with high self-efficacy have strong beliefs to achieve their goals. Therefore, they are seldom affected by negative comments from others, such as stigma. The labeling theory proposed by Link et al. could help us further explain this result, suggesting that stigma affects mental health by destroying the evaluation dimension of self-, concept which is mainly related to self-efficacy [74]. Decreased self-efficacy has been shown to weaken personal empowerment, and reduced power can lead to a higher level of depression [75].

Additionally, Bandura. et al. put forward that self-efficacy relieves depressive symptoms in SUDs patients because depression may stem, in part, from conditions that induce a belief that they cannot overcome the difficulties [76]. Hence, Curran. et al. also explain why self-efficacy can be a strong predictor of abstinence [77]. Earlier studies have shown that self-efficacy can reduce the recurrence rate of substance use patients [78, 79]. We speculate that this may be achieved by reducing depressive symptoms.

These findings emphasize that both stigma and social support should be considered when treating SUDs patients with depression. A prior clinical trial applied acceptance and commitment therapy to reduce the stigma on SUDs patients, and the results showed that decreasing the stigma could improve emotions, cognitions, and behaviors [80]. Psychotherapy research found a relatively enduring and robust effect of stigma on well-being, indicating that if therapists wish to maximize the well-being of the people they treat, they must pay more attention to addressing stigma [81]. The social support network is an essential factor that should be considered in reducing SUDs related stigma [33, 34], which can be subdivided into multiple dimensions according to the source (e.g., peers, family) and type (e.g., general support or specific support for abstinence [82]. For example, peers in mutual aid groups are the primary support source outside of the conventional treatment of alcohol addiction [83]. A clinical study explored the efficacy of 12-step group therapy in 121 patients diagnosed with SUDs and mental disorders showing that self-help groups help reduce mental health and the severity of drug abuse symptoms [84]. Another source of social support is family [85]. Family members, such as parents, play a crucial role in helping patients meet basic demands. Good family support could help patients reduce the impact of stigma [33, 34]. However, if the patient brings a high level of stress and tension, overwhelming the family’s ability to cope, it may lead to reduced family support [86]. Therefore, psychiatrists should pay more attention to increase social support and reduce the stigma of SUDs.

In addition to conventional interventions, doctors should also improve patients’ self-efficacy, reducing the adverse effects of stigma on patients and improving mental health. For example, some research has indicated that applying Zen or Tao can resist the urge to drink or take drugs by enhancing self-efficacy [87]. In addition, psychotherapy research, cognitive-behavioral stress management (CBSM) on self-efficacy and relapses into a form of SUDs, shows that CBSM training contributes positively to increasing self-efficacy and lowering the risks of relapse into once again showing SUDs symptoms [88]. A system review that contained 37 interventions on self-efficacy showed that physical activity interventions might be an excellent choice to enhance self-efficacy [89]. Therefore, when treating SUDs patients with high levels of stigma, clinicians can consider encouraging patients to do more regular physical exercises to improve self-efficacy, reducing the negative emotions of drug patients being affected by stigma.

We should not ignore some limitations in the present research. First of all, the study is a cross-sectional study with some weaknesses, such as the inability to measure the incidence, difficulty making causal inferences, and causal inference [90]. In addition, this study did not control the influence of other confounding variables, for example, whether participants are accompanied by other mental illnesses (e.g., schizophrenia, bipolar disorder, depression, etc.). Moreover, while the model fits patients with SUDs, it is unknown whether the result could be expended to other populations, such as internet addiction.

Despite these limitations, this study contains some strengths. First of all, this is the first study to explore social support mechanisms affecting depression in a large sample of SUDs patients in China. We also consider the moderating effect of self-efficacy in the mediation model, which was ignored in previous studies [44, 72]. Second, our research established a mediation model and chose a more reliable statistic-1000 bootstrapping, to get robust results. Third, this study also provides some advice for clinical psychiatrists to improve treatment effects.

## Conclusion

The present study reported the partial mediating role of stigma in the relationship between perceived social support and depressive symptoms and moderated by self-efficacy among Chinese SUDs patients. The results indicated the critical role of stigma and self-efficacy in treating SUDs patients with depressive symptoms.

## Data Availability

The datasets used and/or analyzed during the current study are available from the corresponding author on reasonable requests.

## Abbreviations

SU-SMS: Substance Use Stigma Mechanisms Scale
MSPSS: The multidimensional scale of perceived social support
CES-D: Centre for Epidemiologic Studies Depression Scale
GSES: General Self Efficacy Scale
SUDs: Substance abuse disorders
CBSM: Cognitive-behavioral stress management
